# Re-initiation of Oral Food Intake Following Enteral Nutrition Alters Oral and Gut Microbiota Communities

**DOI:** 10.3389/fcimb.2019.00434

**Published:** 2019-12-20

**Authors:** Sayaka Katagiri, Takahiko Shiba, Haruka Tohara, Kohei Yamaguchi, Koji Hara, Kazuharu Nakagawa, Keiji Komatsu, Kazuki Watanabe, Yujin Ohsugi, Shogo Maekawa, Takanori Iwata

**Affiliations:** ^1^Department of Periodontology, Graduate School of Medical and Dental Sciences, Tokyo Medical and Dental University, Tokyo, Japan; ^2^Gerodontology and Oral Rehabilitation, Department of Gerontology and Gerodontology, Graduate School of Medical and Dental Sciences, Tokyo Medical and Dental University, Tokyo, Japan

**Keywords:** dysphagia, oral food intake, oral microbiota, gut microbiota, tube feeding

## Abstract

Stroke is associated with multiple forms of disability, including dysphagia. Post-stroke dysphagia increases the risks of pneumonia and mortality and often results in cessation of oral feeding. However, appropriate rehabilitation methods can eventually lead to resumption of oral food intake. This study tried to clarify that re-initiating oral food intake could modify the composition of oral/gut microbial communities in patients with dysphagia. From 78 patients with sub-acute stage of stroke, 11 complete tube feeding subjects without taking antibiotics were enrolled and received rehabilitation for re-initiation of oral food intake, and 8 subjects were brought back to complete oral feeding. Oral and gut microbiota community profiles were evaluated using 16S rRNA sequencing of the saliva and feces samples before and after re-initiation of oral food intake in patients recovering from enteral nutrition under the same nutrient condition. Standard nutrition in the hospital was 1,840 kcal, including protein = 75 g, fat = 45 g, and carbohydrates = 280 g both for tube and oral feeding subjects. Oral food intake increased oral and gut microbiome diversity and altered the composition of the microbiome. Oral and gut microbiome compositions were drastically different; however, the abundance of family *Carnobacteriaceae* and genus *Granulicatella* was increased in both the oral and gut microbiome after re-initiation of oral food intake. Although oral microbiota showed more significant changes than the gut microbiota, metagenome prediction revealed the presence of more differentially enriched pathways in the gut. In addition, simpler co-occurrence networks of oral and gut microbiomes, indicating improved dysbiosis of the microbiome, were observed during oral feeding as compared to that during tube feeding. Oral food intake affects oral and gut microbiomes in patients recovering from enteral nutrition. Rehabilitation for dysphagia can modify systemic health by increasing the diversity and altering the composition and co-occurrence network structure of oral and gut microbial communities.

## Introduction

Stroke is commonly associated with multiple forms of disability, including dysphagia, which is experienced by approximately half the patients in acute phase, 10–20% of patients after 2 weeks, and 11–13% after 6 months (Smithard, 2002). Post-stroke dysphagia also increases the risks of aspiration pneumonia, malnutrition, and mortality (Wade and Hewer, 1987; Axelsson et al., 1989; Kidd et al., 1995). To mitigate these risks, oral food intake is replaced by tube feeding.

Although food delivered through a tube ensures reliable nutrient intake, over the long-term, it can extinguish the pleasure associated with eating and cause gastrointestinal symptoms, such as diarrhea or constipation (Guenter et al., 1991) and micronutrient deficiencies (Reimund et al., 1999). Therapy for post-stroke dysphagia persisting through the convalescence and chronic phases can be combined with diet/eating pattern modifications. Appropriate rehabilitation methods and foods for safe intake are determined based on videofluoroscopy or videoendoscopy (Langmore et al., 1991); continuation of this combined treatment can eventually lead to a resumption of oral food intake.

Many studies have demonstrated that systemic health depends on balanced microbial communities acting synergistically. Dysbiosis of microbiota in the gut can lead to several diseases, including diabetes, rheumatoid arthritis, and inflammatory bowel disease (Honda and Littman, 2012). The gastrointestinal tract begins with the mouth and proceeds to the intestines; ingested bacteria follow this path and thus affect gut microbiota composition.

We speculated that ceasing and re-initiating oral food intake could modify the composition of oral/gut microbial communities in patients with dysphagia. To test this hypothesis, we compared oral and gut microbiome profiles before and after the resumption of oral food intake in patients recovering from enteral nutrition.

## Materials and Methods

### Participants

**Figure 1:**
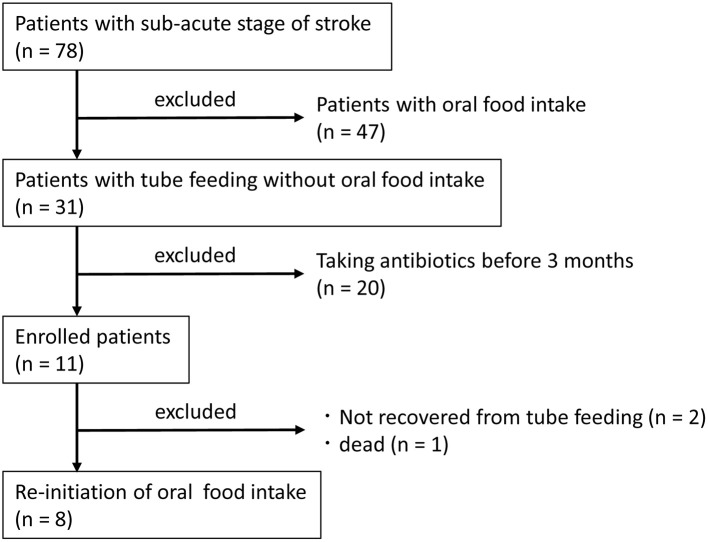
Trial profile.

Patients at Shinyachiyo Rehabilitation Hospital (Chiba, Japan) who were hospitalized between November 2017 and April 2018 and were determined to be in the sub-acute stage of stroke were included in the study. Eligibility criteria were not having an uncontrolled systemic disease and the use of a feeding tube without oral food intake due to dysphagia. Exclusion criteria were treatment with an antibacterial agent within 3 months of disease onset and change in prescription drug use during the study. All participants underwent daily dysphagia rehabilitation with dentists after videofluoroscopy assessment of swallowing function (Baijens et al., 2013). Collecting saliva and feces samples were carried out for each participant at baseline and after 4 weeks of oral food intake without using a feeding tube. Standard nutrition in the hospital was 1,840 kcal, including protein = 75 g, fat = 45 g, and carbohydrates = 280 g both for tube and oral feeding. Seventy-eight subjects were hospitalized in the sub-acute stage of stroke, and 31 subjects were fed entirely by tube (nasogastric or gastrostomy tubes) without oral food intake. Only 11 subjects fulfilled the criteria of the study, and 8 subjects were brought back to complete oral food intake (Figure 1).

The study was registered in UMIN (UMIN000022214) on June 1st, 2016 (https://rctportal.niph.go.jp/en/detail?trial_id=UMIN000022214). The study was carried out in accordance with the Helsinki Declaration of 1975 (revised in 2013) and was approved by our institutional ethics committee (D2015-503). Informed consent was obtained from all participants.

### Evaluation of Swallowing Function

The functional-level of oral food and liquid intake was evaluated with the Functional Oral Intake Scale (FOIS), a 7-point scale (Crary et al., 2005) defined as follows; score 1: can eat nothing by mouth, score 2: tube dependent with minimal attempts of food or liquid, score 3: tube dependent with consistent oral intake of food or liquid, score 4: total oral diet of a single consistency, score 5: total oral diet with multiple consistencies, but requiring special preparation or compensations, score 6: total oral diet with multiple consistencies without special preparation, but with specific food limitations, score 7: total oral diet without no restriction. Japan Coma Scale (JCS) (Shigematsu et al., 2013), which was used to evaluate consciousness, is composed of the following four categories: alert, JCS1 (not fully alert but awake without any stimuli), JCS2 (arousable with stimulation), and JCS3 (unarousable). We measured the levels of C-reactive protein (CRP) as an inflammatory marker and serum albumin (ALB) as a marker of nutritional status. These measurements were carried out for each participant at baseline and after 4 weeks of oral food intake without using a feeding tube.

### 16S rRNA Gene Sequencing and Data Processing

DNA extracted from saliva, and feces samples were purified and used to generate a multiplexed amplicon library (16S rDNA V3–V4 region) as previously described (Komazaki et al., 2017; Sasaki et al., 2018). The MiSeq platform (Illumina, San Diego, CA, USA) was used to generate 250-bp paired-end sequences. Low-quality sequences, pyrosequencing errors, noise, and chimeras were removed. The sequence data have been deposited in the DNA Data Bank of Japan (DDBJ; http://www.ddbj.nig.ac.jp/) under accession number DRA008214. The pre-processed reads were clustered into operational taxonomic units (OTUs) at 97% identity using the CD-HIT-OTU pipeline (http://weizhongli-lab.org/cd-hit-otu/, v.0.01) (Li et al., 2012).

### Evaluation of Microbiome Composition Based on 16S rRNA Sequences and Metagenome Prediction

OTUs were processed and analyzed with the Quantitative Insights into Microbial Ecology (QIIME) v.1.8 software package (Caporaso et al., 2010). Taxonomic classification of 16S rRNA gene sequences at the phylum or genus level was performed using the RDP classifier v.2.2 with default parameters against the GreenGenes database (gg_13_8). Alpha diversity of the samples was measured by the number of OTUs, and the Chao1, and Shannon diversity index (Shannon, 1997). For species-level analyses, we refined taxonomic assignment by BLASTN searches of the DDBJ (as of December 8, 2018). Hits with *E* values ≤ 1e−5 were considered significant. The abundance of 16S rRNA was normalized by converting to reads per million reads (RPM). The PICRUSt v.1.1.3 bioinformatics software package (Langille et al., 2013) was used for metabolic predictions based on closed-reference OTU table generated in QIIME. The OTU table was normalized by dividing the abundances of each OTU by known or predicted 16S rRNA gene copy number abundances. The functional composition of the data was predicted using the Kyoto Encyclopedia of Genes and Genomes (KEGG) database (Kanehisa and Goto, 2000). Predicted pathways in the KEGG database were visualized using iPath3 (Darzi et al., 2018). Dendrograms with heatmaps and a principal coordinate analysis (PCoA) plot were visualized using R v.3.3.2 software. Dissimilarity values (1—Pearson correlation) were clustered using the average linkage method.

### Characterization of Co-occurrence Networks

Co-occurrence coefficients were calculated using the SparCC program (Friedman and Alm, 2012) and 16S rRNA taxonomic abundances, at each stage. Ten iterations were used to estimate the median correlation of each pairwise comparison. The statistical significance of each correlation was calculated by bootstrapping with 500 iterations (Milici et al., 2016). Taxon pairs with SparCC values ≥0.75 were considered as having a co-occurrence relationship with a positive correlation. Our criterion for significance testing was more stringent than the previously used value of ≥0.3 (Shiba et al., 2016). We set the significance levels for the *P* and *q* values to <0.05 and <0.1, respectively. Co-occurrence patterns were drawn using a network structure in which each species and co-occurrence was indicated by a node and edge, respectively, for all species pairs with a positive correlation. We extracted the networks composing of 10 or more nodes from all networks. The networks were visualized using Cytoscape software v.2.8 (Smoot et al., 2011).

### Statistical Analysis

Clinical parameters associated with tube and oral feeding were compared using paired *t*-test (parametric-continuous variables) or Wilcoxon signed-rank test (non-parametric-continuous variable and ordinal variables). Benjamin and Hochberg's false discovery rate was applied for multiple testing, and *q* < 0.1 was considered statistically significant. ANOSIM was used to evaluate the significance of dissimilarity between two groups by applying a dissimilarity matrix value of 1—Pearson coefficient. In the ANOSIM, a *P*-value was obtained by performing a permutation test, which was used to evaluate the statistical significance of the calculated *R*-values.

## Results

### Characteristics of the Study Population

Table 1 shows the characteristics of the study subjects. One subject was fed by a gastrostomy tube, and seven subjects were fed by a nasogastric tube. As shown in the significant increase of FOIS (*P* = 0.009), all subjects in this study could take food through the mouth and recovered from enteral nutrition by the end of the study. CRP level was decreased 4 weeks after re-introduction of oral food intake without using a feeding tube (*P* = 0.018), whereas no changes were observed in body mass index (*P* = 0.544), serum albumin (ALB) (*P* = 0.951), or the JCS (*P* = 1.000) between tube and oral feeding.

**Table 1:** Characteristic of the study population.

**No**	**Age**	**Sex**	**At tube feeding**						**At 4-weeks after re-initiation of oral food intake**					
			**Time after onset of stroke (days)**	**BMI**	**ALB**	**CRP**	**JCS**	**FOIS**	**Time after onset of stroke (days)**	**BMI**	**ALB**	**CRP**	**JCS**	**FOIS**
1	74	F	23	18.8	3.9	0.17	1	1	99	18.3	3	0.16	1	6
2	86	M	40	21.7	3.7	3.54	1	1	106	22.4	3.5	0.44	1	5
3	60	M	33	21.4	3.4	0.6	1	1	80	21.3	3.7	0.2	1	5
4	77	M	28	23.2	3.4	0.1	1	1	116	23.1	3.4	0.06	1	5
5	83	M	60	21.3	3.3	0.19	1	1	117	21.2	3.7	0.02	1	5
6	89	F	50	20.9	3.1	0.13	1	1	80	21.3	3.2	0.01	1	6
7	69	M	57	23.1	3.4	1.77	1	1	170	22.9	4.3	0.31	1	6
8	92	F	45	22.5	3.2	0.09	1	1	122	23.2	2.7	0.09	1	5

*BMI, Body mass index; ALB, serum albumin level; CRP, C-reactive protein; JCS, Japan Coma Scale; FOIS, Functional Oral Intake Scale*.

### Evaluation of Oral and Gut Microbiome Compositions Based on 16S rRNA Gene Sequences

**Figure 2:**
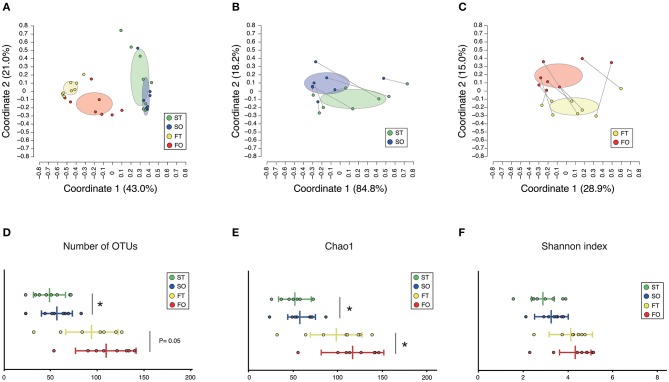
Evaluation of oral and gut microbiome compositions based on 16S rRNA gene sequences between tube and oral feeding (*n* = 8). PCoA analysis **(A)** among oral and gut microbiome between tube and oral feeding, **(B)** in oral microbiome between tube and oral feeding, **(C)** in gut microbiome between tube and oral feeding, **(D)** number of observed species, **(E)** Chao1 index, **(F)** Shannon index among oral and gut microbiome between tube and oral feeding. ST, saliva at tube feeding; SO, saliva at oral feeding; FT, feces at tube feeding; FO, feces at oral feeding. *P < 0.05.

A total of 3,394,127 sequence reads were generated, corresponding to an average of 106,066.5 (range: 70,204–129,901) reads per sample. Rarefaction curves indicated that a sufficient number of reads were obtained for 16S rRNA gene analysis (Supplementary Figure 1). PCoA revealed variations in the microbiome composition of the saliva and feces, indicating that microbial communities in the mouth and gut differed markedly (Figure 2A), as determined by analysis of similarities (ANOSIM) (correlation of *R* = 0.859, *P* = 0.001). Microbiota in the mouth (Figure 2B) and gut (Figure 2C) were altered after resumption of oral feeding, as determined by ANOSIM (saliva, *R* = 0.2327, *P* = 0.035; feces, *R* = 0.2517, *P* = 0.003). In addition, the number of observed OTUs in the mouth and Chao1 index in the mouth and gut were significantly increased following re-initiation of oral food intake; however, the Shannon index of both microbiomes was comparable between tube and oral feeding. The number of species of the gut was increased after the resumption of oral feeding but did not reach the threshold of significance (*P* = 0.05) (Figures 2D–F).

**Figure 3:**
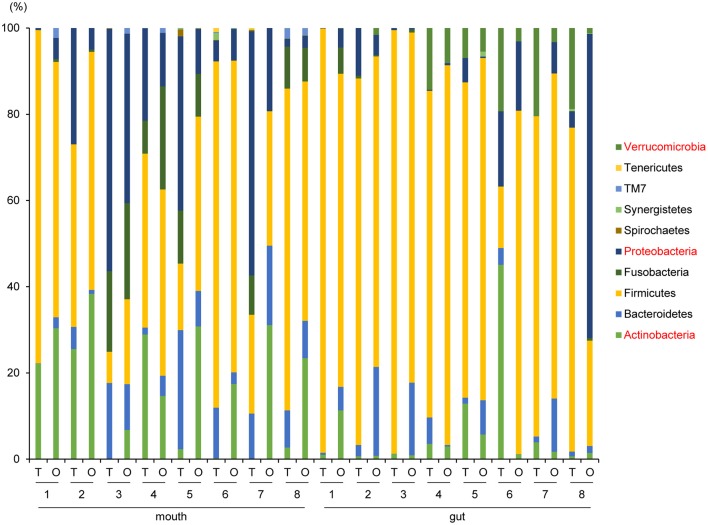
Evaluation of oral and gut microbiome compositions based on 16S rRNA gene sequences between tube and oral feeding at the Phylum level (*n* = 8) (paired *t*-test with FDR). T, tube feeding; O, oral feeding.

Although no significant differences in oral/gut microbiota composition at the phylum level were observed between tube and oral feeding, the abundance of phylum *Actinobacteria* (*P* = 0.03, *q* = 0.26) and *Proteobacteria* (*P* = 0.047, *q* = 0.21) in the mouth tended to increase and decrease, respectively; phylum *Verrucomicrobia* (*P* = 0.049, *q* = 0.39) in the gut showed a decreasing trend after oral feeding (Figure 3).

**Figure 4:**
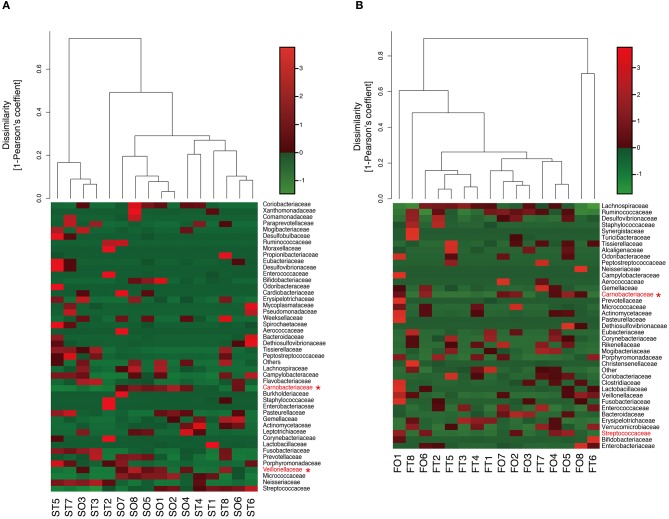
Evaluation of **(A)** oral and **(B)** gut microbiome compositions based on 16S rRNA gene sequences between tube and oral feeding at the Family level (*n* = 8). Dendrogram and heatmap were constructed based on read abundance. *q < 0.1 between tube and oral feeding (paired *t*-test with FDR). ST, saliva at tube feeding; SO, saliva at oral feeding; FT, feces at tube feeding; FO, feces at oral feeding.

**Figure 5:**
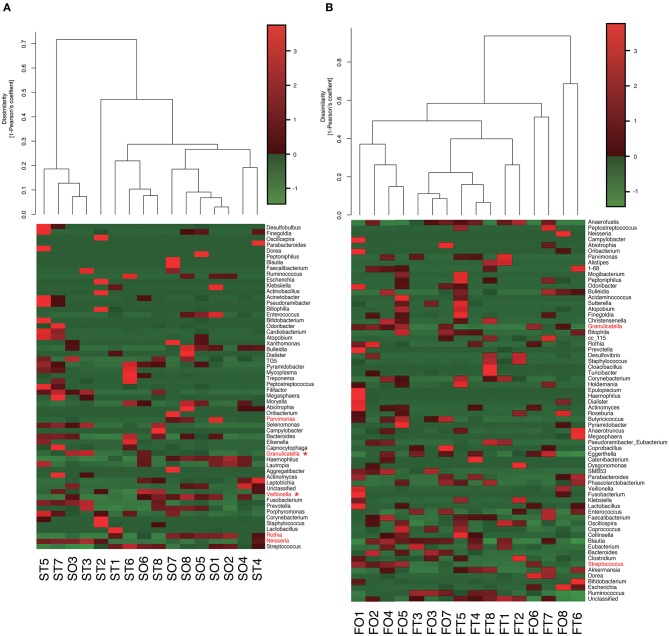
Evaluation of **(A)** oral and **(B)** gut microbiome compositions based on 16S rRNA gene sequences between tube and oral feeding at the Genus level (*n* = 8). Dendrogram and heatmap constructed based on read abundance. *q < 0.1 between tube and oral feeding (paired *t*-test with FDR). ST, saliva at tube feeding; SO, saliva at oral feeding; FT, feces at tube feeding; FO, feces at oral feeding.

**Figure 6:**
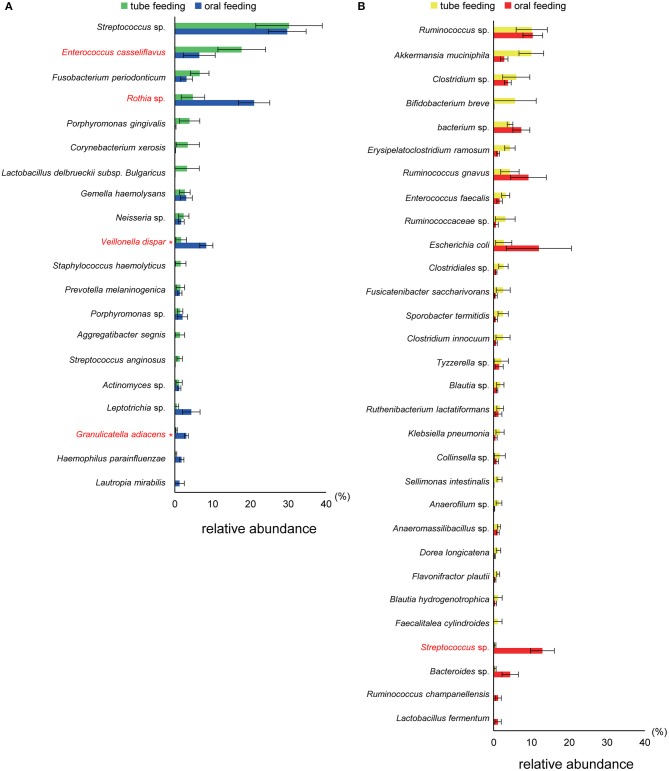
Evaluation of **(A)** oral and **(B)** gut microbiome compositions based on 16S rRNA gene sequences between tube and oral feeding (*n* = 8). Distributions of the species between tube and oral feeding (>1.0% relative abundance). The species name or 16S ribosomal RNA database ID in DDBJ is shown. *q < 0.1 between tube and oral feeding (paired *t*-test with FDR).

Interestingly, the abundance of family *Carnobacteriaceae* was increased in both the mouth (*P* = 0.0009, *q* = 0.02) and gut (*P* = 0.002, *q* = 0.07) after switching from enteral to oral nutrition. *Veillonellaceae* was also significantly overrepresented (*P* = 0.0007, *q* = 0.03) in the mouth after oral feeding. The abundance of family *Streptococcaceae* in the gut tended to increase (*P* = 0.008, *q* = 0.15) after oral food intake (Figures 4A,B). The genera *Veillonella* and *Granulicatella* were significantly overrepresented (*P* = 0.0002, *q* = 0.01 and *P* = 0.0009, *q* = 0.03, respectively) in oral microbiome after oral feeding as compared to tube feeding. Although the abundance of *Granulicatella* was also increased in the gut, this did not attain statistical significance (*P* = 0.002, *q* = 0.12). Genus *Streptococcus* tended to be increase (*P* = 0.008, *q* = 0.26) in the gut at oral food intake. At the genus level, the proportion of *Rothia* (*P* = 0.01, *q* = 0.28) increased whereas those of *Neisseria* (*P* = 0.02, *q* = 0.29) and *Parvimonas* (*P* = 0.03, *q* = 0.34) decreased in the mouth after re-initiation of oral feeding (Figures 5A,B). The most relative abundant species (> 1.0%) in the mouth/gut are shown in Figure 6. *Veillonella dispar* and *Granulicatella adiacens* were highly represented (*P* = 0.001, *q* = 0.07, and *P* = 0.0001, *q* = 0.02, respectively) among oral microbiota after resumption of oral feeding. Although the relative abundance was <1.0%, *G. adiacens* showed an increasing trend (*P* = 0.002, *q* = 0.42) in the gut microbiome after oral feeding; the abundance of *Streptococcus* sp. also increased (*P* = 0.006, *q* = 0.56), although it did not reach statistical significance. In the oral microbiome, the proportion of *Rothia* sp. increased (*P* = 0.02, *q* = 0.78) whereas *Enterococcus casseliflavus* and *Parvimonas* sp. decreased after re-initiation of oral food intake (*P* = 0.02, *q* = 0.64, and *P* = 0.03, *q* = 0.78, respectively). All operational taxonomic units (OTUs) species assignments are shown in Supplementary Table 1.

### Metagenome Prediction of Oral and Gut Microbiomes

**Figure 7:**
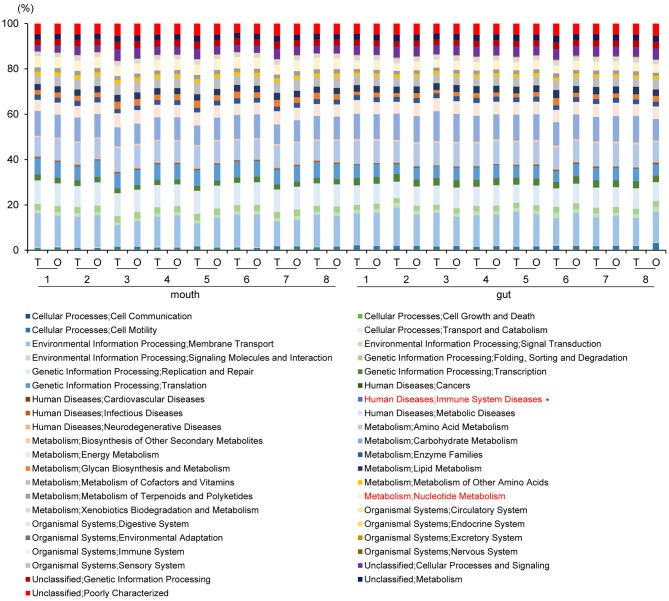
Metagenome prediction at second-level categories among oral and gut microbiome between tube and oral feeding (*n* = 8). *Adjusted *P* < 0.05 between tube and oral feeding (paired *t*-test with FDR). T, tube feeding; O, oral feeding.

PICRUSt analysis yielded predictions of gene function in the oral and gut microbiomes. In the mouth, functional composition with respect to Human Diseases; Immune System Diseases at second-level-categories was significantly decreased (*P* = 0.002, *q* = 0.06). In the gut, an increase of Metabolism; Nucleotide Metabolism (*P* = 0.005, *q* = 0.19) was predicted based on species abundance (Figure 7).

**Figure 8:**
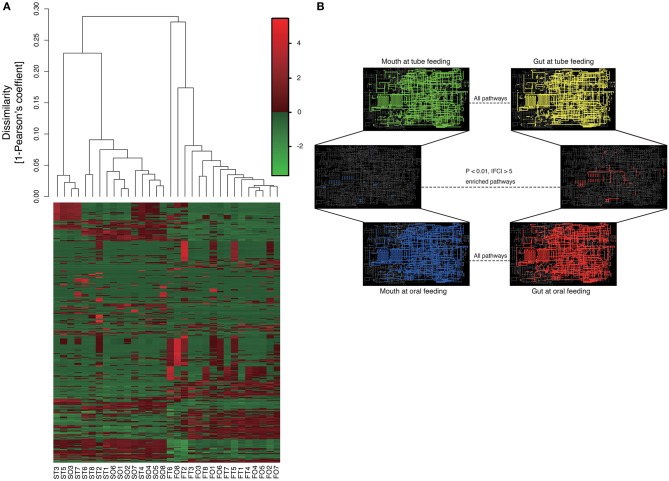
Metagenome prediction at functional-level among oral and gut microbiome between tube and oral feeding (*n* = 8). **(A)** Dendrogram and heatmap constructed based on metagenome prediction. S, saliva; F, feces; T, tube feeding; O, oral feeding. **(B)** Predicted KEGG pathways present in any of samples for oral at tube feeding (upper left), oral at oral feeding (upper right), gut at tube feeding (lower left), and gut at oral feeding (lower right). Middle figure shows enriched (*P* < 0.01, |FC| > 5 pathways. Blue, enriched in mouth; Red, enriched in gut.

Metagenome predictions at functional-level differed markedly between oral and gut microbiomes, as indicated by ANOSIM (*R* = 0.8345, *P* = 0.01), although slight differences were observed after as compared to before resumption of oral feeding within the same sample type (saliva, *R* = 0.1256, *P* = 0.0082; feces, *R* = 0.06083, *P* = 0.149) (Figure 8A and Supplementary Table 2). Interestingly, although the composition of the microbiome at the species level in the gut showed fewer changes than that in the mouth, metagenome prediction in the gut showed more enriched pathways (*P* < 0.01, |fold change| > 5). Fatty acid biosynthesis and degradation were enriched in both the oral and gut profiles (Figure 8B).

### Network Analysis in Oral and Gut Microbiota

**Figure 9:**
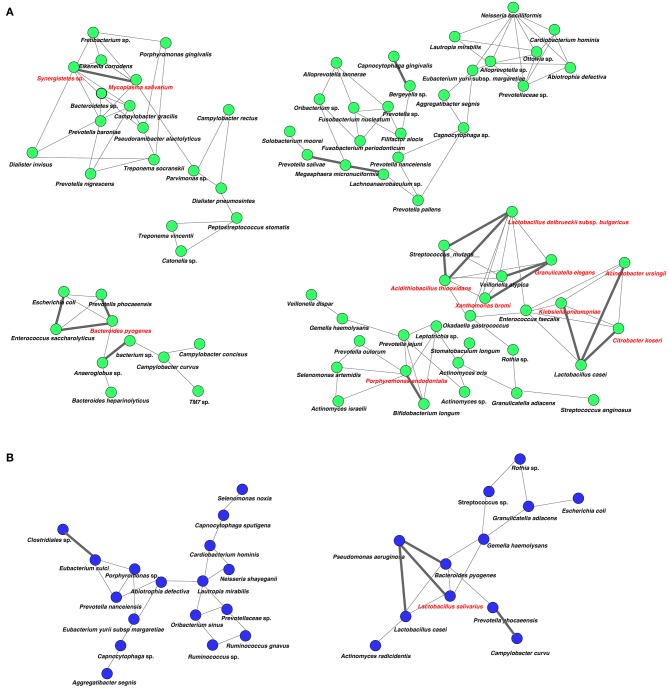
Co-occurrence network in the oral microbiome. All networks are shown with each species and co-occurrence relationship indicated by a node and edges, respectively. Interactions with significant co-occurrence network are indicated with bold lines. Interacting core species (showed co-occurrence with other species ≥ 4 and significant co-occurrence ≥ 1 in main networks) are indicated in red text. **(A)** Tube feeding, **(B)** oral feeding.

**Figure 10:**
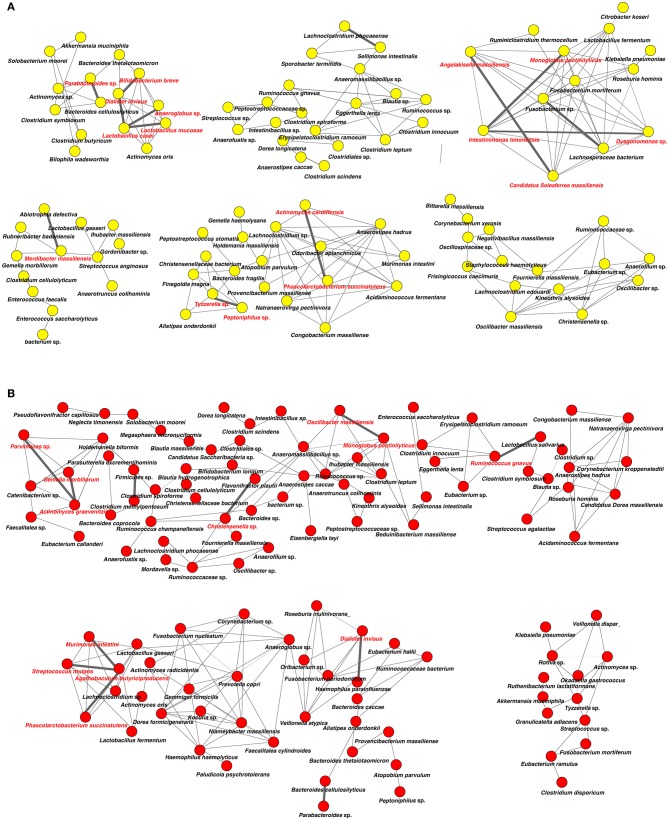
Co-occurrence network in the gut microbiome. All networks are shown with each species and co-occurrence relationship indicated by a node and edges, respectively. Interactions with significant co-occurrence network are indicated with bold lines. Interacting core species (showed co-occurrence with other species ≥ 4 and significant co-occurrence ≥ 1 in main networks) are indicated in red text. **(A)** Tube feeding, **(B)** oral feeding.

Analysis of specific microbial co-occurrence patterns is a useful tool to reveal various biological contexts (Stuart et al., 2003). We analyzed co-occurrence relationships in 16S rRNA profiles of species by constructing network structures (referred to as co-occurrence networks) in which two co-occurring species were indicated by nodes and connected by a degree. The values of clustering coefficients in all networks were decreased from 0.447 to 0.375 in the oral microbiome between tube and oral feeding, indicating a shift toward simpler microbial networks after oral feeding. The number of main networks that showed 10 or more nodes were decreased from 4 to 2 in the oral microbiome when comparing tube and oral feeding. Focusing on the main networks, average connected degrees per node were 1.91 and 1.28, and total numbers of nodes were 70 and 29 in oral microbiome in tube and oral feeding, respectively (Figures 9A,B). In the gut microbiome, the values of clustering coefficients in all networks were also decreased from 0.542 to 0.467 between tube and oral feeding. The number of main networks that showed 10 or more nodes were also decreased from 6 to 3 in the gut microbiome after oral feeding. The average values of connected degrees per node were 2.07 and 1.75, and the total numbers of nodes were 96 and 118 in the gut microbiome with tube and oral feeding, respectively (Figures 10A,B). The species with four or more co-occurrence with other species and one or more significant co-occurrence in the main networks were classified as interacting core species. Interacting core species were altered by re-initiation of oral food intake both in oral and gut microbiomes. The number of interacting core species was decreased from 11 to 1 in oral and from 12 to 9 in the gut.

## Discussion

In this study, we tried to evaluate how oral food intake itself affects oral and gut microbiota under the same nutrient conditions. The results of this study demonstrate that oral food intake alters oral and gut microbiota community composition and abundance in patients recovering from enteral nutrition. After resumption of oral feeding, the diversity of both microbiomes were increased. Gut microbiota diversity is essential for maintaining health. Loss of species richness in microbiome is associated with dysbiosis (Levy et al., 2017). We also observed significant differences between oral and gut microbiota communities, which is consistent with previous findings (Aagaard et al., 2014).

Interestingly, reinstatement of oral food intake affected some of the same taxa in the oral and gut microbiomes. For example, family *Carnobacteriaceae* and genus *Granulicatella* were overrepresented in both oral and gut profiles following re-initiation of oral feeding, suggesting that ingested oral bacteria may directly affect the gut microbial community. *Carnobacteriaceae* belongs to the *Lactobacillales* order and may be associated with hormone production (Antwis et al., 2019). *Granulicatella* is mouth- and gut-resident, catalase- and oxidase-negative, facultative anaerobic, Gram-positive coccus belonging to *Carnobacteriaceae* (Ludwig et al., 2009). Some *Granulicatella* species are known to cause infection (Cargill et al., 2012), whereas *Granulicatella* biofilms contribute to human gingiva epithelial barrier function (Shang et al., 2018). In this study, the proportion of *G. adiacens* in the oral/gut microbiome was increased by re-initiation of oral food intake. Although no change was observed in the gut, family *Veillonellaceae* was highly overrepresented in the mouth after oral food intake. Oral *Veillonella*, including *Veillonella dispar*, are known as early colonizers in oral biofilm formation and show several interactions with *Streptococcus* and *Veillonella* species (Mashima and Nakazawa, 2014). Interestingly, the abundance of family *Streptococcaceae*, genus S*treptococcus*, and S*treptococcus* spp., which belong to the core microbiota of the mouth-resident bacterium (Chalmers et al., 2008), was dramatically increased (>30-fold) in the gut after oral feeding, although this did not reach statistical significance. These results provide further evidence that oral microbiota may directly affect those in the gut and suggest that oral bacteria could affect systemic health through modulation of gut microbiota.

In this study, increases in the abundance of phylum *Actinobacteria*, genus *Rothia* and *Rothia* sp., and decreases in that of genus *Parvimonas, Parvimonas* sp., genus *Neisseria*, and *E. casseliflavus* in the mouth after re-initiation of oral feeding were observed. *Rothia* spp., which are Gram-positive bacteria residing in the human oral cavity and pharynx (Tsuzukibashi et al., 2017), showed an almost 4-fold increase. Interestingly, *Actinobacteria* is less abundant in the oral microbiome of patients with diabetes as compared to healthy controls, and oral *Actinobacteria* is associated with type 2 diabetes (Long et al., 2017). On the other hand, the levels of some pathogenic bacteria showed a decreasing trend. *Parvimonas* sp. is detected in periodontal-endodontic lesions (Li et al., 2014), and although in most cases *Neisseria* species are benign commensals present in the oral and nasopharyngeal cavities of their human host, their presence and abundance have been correlated with the onset and progression of many diseases (Liu et al., 2015). However, there is a possibility that oral *Neisseria* spp. (e.g., *N. subflava*) may not be the same as the *Neisseria* spp. found in diseases (e.g., *N. meningitidis*). In addition, while genus *Enterococcus* generally exists in the gut as a commensal microorganism, *E. casseliflavus* is known to cause infection (Fluit et al., 2001). A decreased abundance of *E. casseliflavus* may be beneficial for overall health in the mouth, and we previously reported that mice gavaged with the periodontal pathogenic bacterium *Aggregatibacter actinomycetemcomitans* showed impaired glucose tolerance, liver steatosis, and changes in the gut microbiome (Komazaki et al., 2017). In addition, dysbiosis of oral microbiota can lead to oral disease and impair innate host defenses (Lamont et al., 2018).

The metagenome prediction based on 16S rRNA genes in oral microbiota revealed the downregulation of genes related to immune system disease. This finding may be explained by a decreased abundance of pathogenic bacteria. The upregulation of genes involved in nucleotide metabolism in the gut may promote food digestion. The ANOSIM results showed that gene function profiles of oral and gut microbiomes did not change so much after the resumption of oral feeding. The concept of functional redundancy is at the core of the theory relating changes in ecosystem function to species loss. Functional redundancy is based on the observation that some species perform similar roles in communities and ecosystems and may, therefore, be substitutable with little impact on ecosystem processes (Lawton and Brown, 1993). Although more apparent changes were observed for oral than for gut microbiota composition after re-initiation of oral food intake, the metagenome prediction showed more differentially enriched pathways in the gut, especially those related to fatty acid metabolism.

Surprisingly, microbial network in oral/gut microbiome was dramatically changed after re-initiation of oral food intake. Both networks in microbiomes appeared to be simpler, and the number of networks decreased by re-initiation of oral food intake. Overall, these phenomena may indicate that networks were restructured to more closely resemble a healthy condition. In addition, alteration of interaction between core species may suggest an improvement of microbiome dysbiosis. After the re-initiation of oral food intake, only *Lactobacillus salivarius* was defined as an interacting core species in the oral microbiome. The possibility that *L. salivarius* could inhibit colonization of *Helicobacter pylori* in the stomach was reported (Kabir et al., 1997). Furthermore, the administration of *L. salivarius* could prevent dental caries and periodontitis (Higuchi et al., 2019). On the other hand, *Christensenella* sp. was identified as an interaction core species in the gut microbiome after oral feeding. Family *Christensenellaceae* was influenced by the host genetics, and obese-associated microbiome was rescued by transplanted *Christensenella minuta* in mice (Goodrich et al., 2014).

Early enteral feeding is effective in dysphagic stroke patients for whom oral intake is deemed inadvisable. Proper tube feeding decreases the risk of aspiration pneumonia, malnutrition, and dehydration. However, the rate of dysphagia in stroke patients decreases over time (Smithard, 2002), and many chronic stroke patients can take in food through the oral route (Nakajima et al., 2014). In this study, the swallowing function of post-stroke patients with dysphagia was assessed by videofluoroscopy or videoendoscopy, and adequate rehabilitation methods and the level of the dysphagia diet were determined. By following these recommendations, all patients were capable of oral food intake by the end of the study.

Langdon et al. reported that tube-fed survivors experienced significantly higher rates of respiratory infections than survivors fed orally in 330 ischemic stroke survivors (Langdon et al., 2009). While essential for enjoying meals, oral food intake may also play an important role in preventing respiratory infections such as pneumonia. Several studies have reported that orally fed patients have higher activity of daily living (Nakayama et al., 2014) and quality of life (Hong and Yoo, 2017) than tube-fed patients, highlighting the physiological and psychosocial benefits of oral food intake. In previous studies comparing the oral flora of patients fed via the two routes, the proportion of pathological bacteria such as *Streptococcus agalactiae* was increased in patients fed through a tube (Leibovitz et al., 2003; Takeshita et al., 2011). Two main factors could affect oral flora community composition. First, oral clearance may be diminished in seniors due to aging and medication, allowing pathological bacteria to proliferate in the mouth (Palmer et al., 2001). Secondly, it could depend on whether the patient masticates the food. In this study, none of the subjects were taking antibiotics or changed their medication. Importantly, nutrient levels, including protein, fat, and carbohydrate, and caloric intake did not differ between tube and oral feeding. These results demonstrate that oral feeding can alter the oral as well as the gut microbiome. However, in the present study, adequate amount of fiber based on the nutrient criteria was supplied to the participants at oral feeding in the hospital-prepared food. On the other hand, enteral nutrients provided by medical insurance in Japan contain extremely low dietary fiber. Thus, we consider that the total amount dietary fiber at oral feeding was higher than that in tube-fed participants in the present study. Given that dietary fiber has a positive effect on microbiota and its metabolism in the gut (Rao et al., 1994), the result from the present study might partially be due to the higher amount of dietary fiber at oral feeding. The self-cleaning ability of the oropharynx was preserved in patients who were able to take food orally, as this act promoted saliva secretion as the food bolus passed through the mouth during mastication. In a study of 331 patients hospitalized for aspiration pneumonia, those who were prohibited from oral food intake had a more extended treatment period and poor swallowing function compared to those who were able to eat food at an early stage of hospitalization (Maeda et al., 2016). In addition, long-term intravenous and enteral nutrition management can have adverse consequences such as gastrointestinal symptoms (Guenter et al., 1991), diarrhea, constipation and gastroesophageal reflux disease, and deficiency of trace elements (Reimund et al., 1999).

In conclusion, oral food intake affects mouth and gut microbiomes in patients recovering from enteral nutrition. Rehabilitation for dysphagia can modify oral and gut microbiota communities and improve swallowing function. To the best of our knowledge, this is the first study to demonstrate the importance of food intake by the oral route from the viewpoint of microbiology.

## Data Availability Statement

The datasets generated for this study can be found in the DNA Data Bank of Japan (DDBJ)/DRA008214.

## Ethics Statement

The studies involving human participants were reviewed and approved by Tokyo Medical and Dental University ethics committee. The patients/participants provided their written informed consent to participate in this study.

## Author Contributions

SK and TS performed most of the experiments and wrote the first draft of the manuscript. HT, KY, KH, KN, KK, KW, YO, SM, and TI assisted in some studies and reviewed the manuscript. HT, KY, KH, and KN performed rehabilitation for dysphagia and collected samples. TS and KK provided expertise on microbiome analysis. SK and HT supervised all the studies and the writing of the manuscript.

### Conflict of Interest

The authors declare that the research was conducted in the absence of any commercial or financial relationships that could be construed as a potential conflict of interest.
