# Supplementary figures and images for: Re-initiation of Oral Food Intake Following Enteral Nutrition Alters Oral and Gut Microbiota Communities
Source: Front Cell Infect Microbiol. 2019 Dec 20;9:434. doi: 10.3389/fcimb.2019.00434 (PMC6951430; doi:10.3389/fcimb.2019.00434)

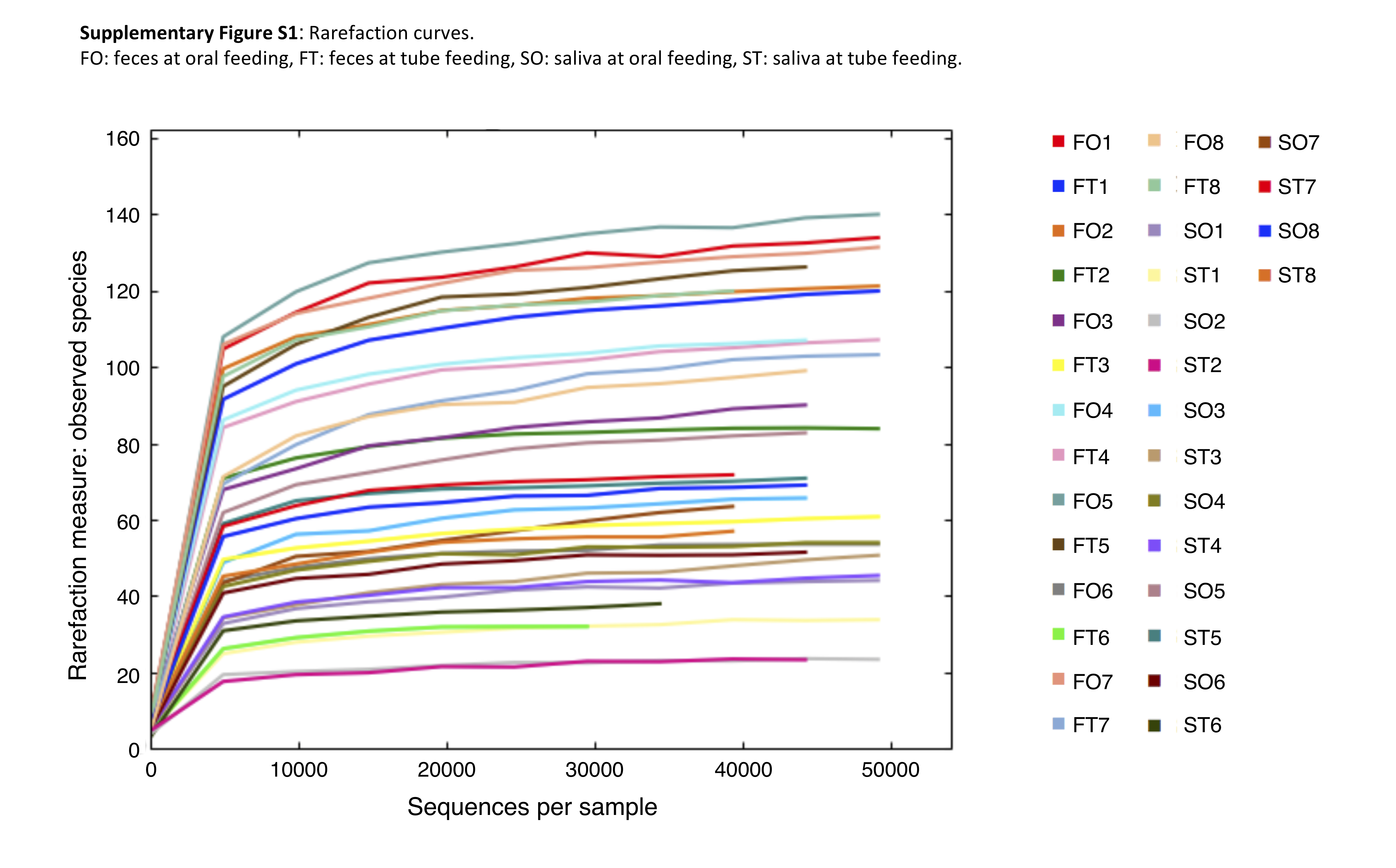

Supplement: Supplementary file 3 [file Image_1.JPEG]
